# Malaria transmission potential could be reduced with current and future climate change

**DOI:** 10.1038/srep27771

**Published:** 2016-06-21

**Authors:** C. C. Murdock, E. D. Sternberg, M. B. Thomas

**Affiliations:** 1 Department of Infectious Diseases, College of Veterinary Medicine, Odum School of Ecology, University of Georgia, 260 Veterinary Medicine, 501 D.W. Brookes Drive, Athens GA 30602, USA; 2 Center for Infectious Disease Dynamics, Department of Entomology, Pennsylvania State University, Merkle Lab, Orchard Road, University Park, PA 16802, USA

## Abstract

Several studies suggest the potential for climate change to increase malaria incidence in cooler, marginal transmission environments. However, the effect of increasing temperature in warmer regions where conditions currently support endemic transmission has received less attention. We investigate how increases in temperature from optimal conditions (27 °C to 30 °C and 33 °C) interact with realistic diurnal temperature ranges (DTR: ± 0 °C, 3 °C, and 4.5 °C) to affect the ability of key vector species from Africa and Asia (*Anopheles gambiae* and *An. stephensi*) to transmit the human malaria parasite, *Plasmodium falciparum.* The effects of increasing temperature and DTR on parasite prevalence, parasite intensity, and mosquito mortality decreased overall vectorial capacity for both mosquito species. Increases of 3 °C from 27 °C reduced vectorial capacity by 51–89% depending on species and DTR, with increases in DTR alone potentially halving transmission. At 33 °C, transmission potential was further reduced for *An. stephensi* and blocked completely in *An. gambiae*. These results suggest that small shifts in temperature could play a substantial role in malaria transmission dynamics, yet few empirical or modeling studies consider such effects. They further suggest that rather than increase risk, current and future warming could reduce transmission potential in existing high transmission settings.

The dynamics and distribution of human malaria strongly depend on the interplay between the parasite, the mosquito vectors and the environment^1^. The key parasite and mosquito life history traits that determine transmission intensity exhibit clear, non-linear relationships with temperature^2^. This non-linearity means that small changes in environmental temperature can lead to large changes in transmission risk. Recent evidence suggests that malaria incidence is increasing in cooler regions of the world due to global warming^3^,^4^,^5^,^6^,^7^,^8^. However, the effects of environmental change on malaria transmission are potentially complex^5^,^6^,^7^,^9^, and the implications for malaria risk in optimum transmission settings remain poorly defined. Additionally, irrespective of climate change, mosquitoes are exposed to a range of microclimates due to variation in local habitat features, which affect mean ambient temperature and diurnal temperature range (DTR)^10^,^11^. Here we investigate how shifts in temperature around optimum conditions representative of endemic transmission settings, affects the capacity of two key vector species - *Anopheles gambiae* from Africa and *An. stephensi* from Asia - to transmit the human malaria parasite *Plasmodium falciparum*.

To examine the effects of temperature on vectorial capacity, we provided female mosquitoes with either an uninfected or *P. falciparum*-infectious bloodmeal and then randomly placed them across three mean temperatures. These mean temperatures were 27 °C, representing conditions typically considered optimal for mosquito and parasite development, and 30 and 33 °C, representing warmer environments within the current transmission range and/or future environments after projected climate warming^1^. We also included three diurnal temperature ranges (DTR of 0, 6 and 9 °C) for each mean temperature, because temperatures are not constant in nature and daily cycles in temperature can shape insect and parasite life history traits beyond the effect of mean temperature alone^6^,^12^. We then quantified the effects of mean ambient temperature and DTR on measures of vector competence, such as parasite prevalence at different developmental stages (proportion of mosquitoes with oocysts or sporozoites) and parasite intensity (number of oocysts/midgut), as well as daily mosquito survival. To assess the effects of variation in mean temperature and DTR on overall transmission risk, we used these data to estimate changes in vectorial capacity, a standard metric used to define the transmission potential of a vector population.

For both *An. gambiae* and *An. stephensi*, increases in mean ambient temperature resulted in significant decreases in oocyst prevalence, oocyst intensity, and sporozoite prevalence (Table 1; Fig. 1, SI Table S1). These results suggest that mean temperatures above 27 °C are less permissive for parasite establishment and development. The effects of warmer temperatures on vector competence were much more pronounced in *An. gambiae* than in *An. stephensi*. The effect of varying DTR was mixed and, again, dependent on vector species. For example, increasing DTR had a marginally significant effect on oocyst prevalence (Fig. 1A), and no effect on oocyst intensity (Fig. 1C) or sporozoite prevalence (Fig. 1E) in *An. gambiae* (Table 1). In *An. stephensi*, increasing diurnal temperature fluctuation also significantly reduced oocyst prevalence (Fig. 1B) and intensity (Fig. 1D, SI Table S2). However, the effect of DTR on oocyst intensity only occurred when *An. stephensi* were housed at a mean 27 °C (Fig. 1D, SI Table S2). Unexpectedly, the impact of DTR on sporozoite prevalence was marginally non-significant, despite effects of variation in DTR on oocyst establishment in *An. stephensi* (Table 1, Fig. 1F).

Increasing mean ambient temperatures significantly reduced daily mosquito survival for both *An. gambiae* and *An. stephensi* (SI Table S3, Fig. 2), and these effects were again more marked in *An. gambaie*. *An. stephensi* experienced higher daily survival across all treatment groups throughout the course of each experiment relative to *An. gambiae* (Fig. 2). There were also significant main effects of variation in DTR on adult longevity (SI Table S3); however, the effect was dependent upon mean ambient temperature (SI Table S3) and was qualitatively different for each vector species (SI Table S4). For example, in *An. gambiae*, modest diurnal temperature fluctuation (DTR 6 °C) around the hottest mean temperature (33 °C) buffered mosquitoes from the effects of hot mean temperatures on daily mosquito survival, while more extreme temperature fluctuation (DTR 9 °C) exacerbates these effects relative to mosquitoes housed in a thermally constant, hot environment (Fig. 2A). In *An. stephensi*, diurnal temperature fluctuation in general decreased daily mosquito survival only in mosquitoes housed at a mean 27 °C (Fig. 2B).

Whether or not mosquitoes were infected with *P. falciparum* significantly affected the daily survival probability for both *An. gambiae* and *An. stephensi* (SI Table S3), although the effects again were qualitatively different for each mosquito species (SI Fig. S1, SI Table S5). *P. falciparum* infection in *An. gambaie* increased mosquito survival at extreme ambient temperatures relative to bloodfed controls. In contrast, *P. falciparum* infection significantly decreased *An. stephensi* survival in constant temperature treatments only (SI Fig. S1, Table S5). The species-specific effects of ambient temperature and DTR on mosquito survival illustrate complex ‘mosquito x environment’ interactions, which might partially explain the mixed evidence for fitness effects of malaria infection on mosquitoes, with the majority of studies reporting negative or no effects reviewed in^13^, and only one reporting positive effects of infection on mosquito survival^14^.

The effects of increasing mean ambient temperatures and DTR on individual vector-parasite traits translate into dramatic reductions in overall vectorial capacity for both *An. gambiae* and *An. stephensi*, with the impact most pronounced for *An. gambiae* (Fig. 3A). When rate summation was used to predict additional effects of DTR on the extrinsic incubation period of the parasite and daily mosquito biting rates, we see even further reductions in vectorial capacity in mosquitoes housed at all temperatures (Fig. 3B).

We demonstrate that relatively small increases in mean temperature and diurnal temperature range around the temperature optimum can both lead to marked reductions in vectorial capacity. For example, an increase of 3 °C from a highly permissive temperature of 27 °C reduced vectorial capacity of *An. stephensi* by 51–66%. For *An. gambiae* the effects were even greater, with vectorial capacity reduced by as much as 84–89%, depending on DTR. This also means that a decrease in temperature from 30 to 27 °C (as might occur seasonally) could increase transmission potential by 629–814%, depending on DTR. Furthermore, increases in DTR alone could halve vectorial capacity. The effects of DTR are highly relevant to ongoing changes in housing design that are taking place in many parts of Africa^15^. The transition from traditional materials such as mud and thatch to modern brick and metal alters indoor microclimate and can increase DTR by 2–5 °C (SI Fig. S2). Such changes to indoor climate could yield almost instantaneous reductions in vectorial capacity of highly endophilic vectors such as *An. gambiae.* Note that in the current study our estimates of vectorial capacity assume all blood meals are taken from human hosts. In reality, mosquitoes can exhibit different levels of anthropophagy depending on species and local host diversity. *Anopheles stephensi*, for example, is known to be strongly zoophilic^16^ and this is likely to reduce absolute values of vectorial capacity in nature. Nonetheless, the relative changes in vectorial capacity we observe should remain unchanged.

In our experiments, mosquito larvae were maintained under standard insectary conditions before being transferred to the different temperature treatments as adults. This approach reflects the fact that temperatures in natural larval habitats can differ substantially from ambient air temperatures experienced by adult mosquitoes^17^, and that adults emerging from a common larval habitat can potentially distribute across diverse local microclimates^10^. Nonetheless, we acknowledge that temperature variation can also impact larval life history traits^18^, and that temperature effects can integrate across life stages, potentially exacerbating the impact of climate warming^19^. How such effects play out in the field could be further shaped by variations in biotic factors^20^, genotypic differences between local mosquito and parasite populations^21^ and mosquito behavior^14^ (although there is limited evidence to support precise behavioral thermoregulation in malaria mosquitoes^22^,^23^). Transmission intensity and ultimately disease burden are also strongly determined by rainfall, control measures and socio-economic factors^1^. Even so, direct effects of temperature on mosquito life history and malaria parasites remain important determinants of disease risk^24^,^25^,^26^,^27^.

The laboratory colonies of mosquitoes that we used are likely adapted to standard insectary temperatures of 25–27 °C, and the parasite strain has also been selected to infect optimally under these conditions. In nature, local adaptation could lead to different temperature optima, and different susceptibilities to temperature fluctuation^21^. Such local adaptation could limit direct extrapolation of our results to field settings. However, there is scant information on the nature and extent of local adaptation in malaria vectors^21^, and mean temperatures of 25–27 °C are typical for areas with high endemic transmission^28^. One of the only studies to explore local thermal adaptation in a mosquito vector (in this case *Culex pipiens*, a vector of certain arboviruses) found that although local populations differed in key life history traits such as development and survival, variation was not correlated with local temperatures and thus did not support the local thermal adaptation hypothesis^29^.

Regardless of local adaptation, the unimodal, nonlinear relationships between life history traits and temperature are fundamental^30^. Moreover, upper critical temperatures exhibit limited capacity for response to selection^31^,^32^, suggesting that effects of exposure to high temperatures tend to be conserved at the species level. This premise is supported by the fact that our two mosquito species did not exhibit identical responses to high temperatures, even though they have been under similar lab-based selection for many generations. Thus, we expect variation in temperature above the optimum to yield reductions in transmission potential irrespective of local adaptation. The more general caution regarding extrapolation of lab-based results to field settings is important to acknowledge. However, data on the effects of temperature on malaria mosquito and parasite traits are surprisingly scant. For example, the best available data to describe the relationship between vector competence and temperature^2^ derive from a single, poorly replicated study published in 1940 that actually examined *P. vivax* infection in a North American vector species^33^. Certain modeling studies ignore temperature dependence of traits such as competence, in part, because of the scant nature of the data^9^,^28^. We believe our study provides important new insights into the temperature dependence of malaria transmission that should motivate further field research.

The Intergovernmental Panel on Climate Change 2014^8^ predicts global warming to cause expansions in the current geographical range of malaria and other vector-borne diseases, due to increases in suitable habitat for vectors and increased duration of the transmission season. The current study provides important empirical evidence to counterbalance this recurrent emphasis in the climate change literature. Based on data in the Malaria Atlas Project (http://www.map.ox.ac.uk/), we estimate there are >320 million people at risk in the highest transmission areas of Africa (i.e. areas with optimal conditions for endemic transmission leading to annual *P. falciparum* parasite rates in the 2–10 year old age class >40%). In high transmission settings, the epidemiology of malaria is complex, not least because prevalence exhibits a strongly non-linear, saturating relationship with measures of transmission intensity such as vectorial capacity or entomological inoculation rate^34^. Thus, changes in transmission potential need not lead to obvious changes in prevalence, although the link to incidence is likely more direct. Nonetheless, changes in temperature due to modification of local landscape and housing, or longer-term climate change, could work in concert with control efforts and improvements in public health infrastructure to reduce malaria transmission in these highly endemic settings. Indeed, there is some evidence that this might already be occurring^35^.

At a more basic level, our data emphasize the importance of local environmental context for understanding temporal and spatial patterns of transmission. We demonstrate multi-fold-differences in malaria transmission potential due to small changes in mean temperature and DTR. Transient shifts of ±3 °C or more in mean temperature and DTR are commonplace (SI Fig. S2 6,10) and do not require long-term climate change to be relevant, yet few studies consider such effects. We also demonstrate important differences between vector species in thermal sensitivity of life history traits and overall vector competence, cautioning against the use of mixed-species data and extrapolation across vector-parasite pairings, which is a common feature of many studies exploring environmental influences on transmission^2^,^7^,^9^,^28^.

## Methods

We reared *Anopheles gambiae* (Keele strain) and *Anopheles stephensi* (Liston) under standard insectary conditions at 27 ± 0.5 °C, 80% humidity, and a 12 h light: 12 h dark photo-period and on a 10% glucose solution diet. Upon emergence, three-day old female adult mosquitoes were randomly distributed into experimental cages (20 × 20 × 20 cm; N = 150) representing one of 18 treatment groups consisting of three mean temperatures (*27* °*C*, *30* °*C* and *33* °*C*), three diurnal temperature ranges (*DTR 0* °*C*, ±*0* °*C*; *DTR 6* °*C*, ±*3* °*C*; and *DTR 9* °*C*, ±*4.5* °*C*), and two infection treatments (bloodfed controls and *P. falciparum* infected) (SI Fig. S3). We have two and three full biological replicates of the *An. gambiae* and *An. stephensi* experiments, respectively. Mosquitoes were deprived of sugar for a 12 hr interval of time, after which they received either an uninfected bloodmeal or a *Plasmodium falciparum* (8% gametocytemia; NF54 isolate, MR4) infectious bloodmeal through a membrane feeder. Both *An. gambiae* and *An. stephensi* received an infectious bloodmeal from the same *P. falciparum* culture to ensure similar parasite dosages and minimize any inter-culture variation. Directly after the bloodfeeds, mosquitoes were then placed into the appropriate temperature treatment and were maintained on 10% glucose solution daily.

Mean temperatures and diurnal temperature ranges were selected based on microclimate data collected from various housing types throughout the transmission season in Chennai, India^11^ and Tanzania^15^, and we used the Parton-Logan model for diurnal temperature fluctuation (SI Methods) to program our fluctuating reach-in incubators. We dissected midguts and salivary glands on days 7 and 15 post-infection (PI) from each *P. falciparum* exposed treatment group to quantify the effects of variation in mean temperature, diurnal temperature fluctuation, and treatment on measures of vector competence (SI Methods). Throughout the duration of the experiment we counted the number of dead mosquitoes in each cage daily to quantify the effects of mean temperature, diurnal temperature fluctuation, and treatment on daily mosquito mortality.

All statistical analyses for these experiments were run in IBM SPSS Statistics 22.0 (IBM Corporation). We used mixed effects generalized linear models to assess the effects of temperature, diurnal temperature range, and infection treatment on the following response variables: oocyst prevalence, oocyst intensity, sporozoite prevalence, and daily mosquito survival. Temperature (27 °C, 30 °C, and 33 °C), diurnal temperature range (DTR 0 °C, DTR 6 °C, and DTR 9 °C), and their interaction were included in all models as fixed effects. Infection treatment (bloodfed control and *P. falciparum* infected) and days post-infection were included in all survival models as an additional fixed effect and a covariate, respectively. To control for any variation influencing our response variables across biological replicates, we included replicate as a random factor in all model analyses (SI Methods).

We calculated vectorial capacity (*C*) with the following equation:


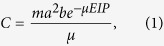


where *m* represents infectious vector density, *a* is the daily probability of a human host being fed on by a vector, *EIP* is the extrinsic incubation period of the parasite, *μ* is the daily probability of adult mosquito mortality, and *b* is vector competence. The density of infectious mosquitoes (*m*) was estimated by taking the average number of mosquitoes alive in each treatment group upon completion of the parasite’s extrinsic incubation period at each temperature. Due to significant block effects, we used estimated marginal means from our mixed model analysis to parameterize vector competence (*b*; the proportion of infectious mosquitoes), and the daily probability of mortality of potentially infectious adult mosquitoes (*μ*) for each treatment group. To estimate the parasite extrinsic incubation period (*EIP*) and mosquito biting rate (*a*) at a given mean temperature (*T*), we used Briere’s thermal equation:


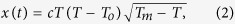


where *T*_*o*_ and *T*_*m*_ are the thermal minimum and maximum for a given trait (*x*), and *c* is a constant. The values used for *T*_*o*_, *T*_*m*_, and *c* for the extrinsic incubation period (*EIP*) and the daily biting rate (*a*) were taken from Mordecai *et al*.^2^. In order to estimate potential effects of diurnal temperature fluctuation on these parameters, we used rate summation^34^ defined as


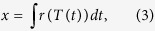


where a given trait (*x*) is defined as a rate (*r*) that adjusts instantaneously to temperature (*T*), which in turn is a function of time (*t*).

## Additional Information

**How to cite this article**: Murdock, C. C. *et al*. Malaria transmission potential could be reduced with current and future climate change. *Sci. Rep.* **6**, 27771; doi: 10.1038/srep27771 (2016).

## Supplementary Material

Supplementary Information

## Figures and Tables

**Figure 1:**
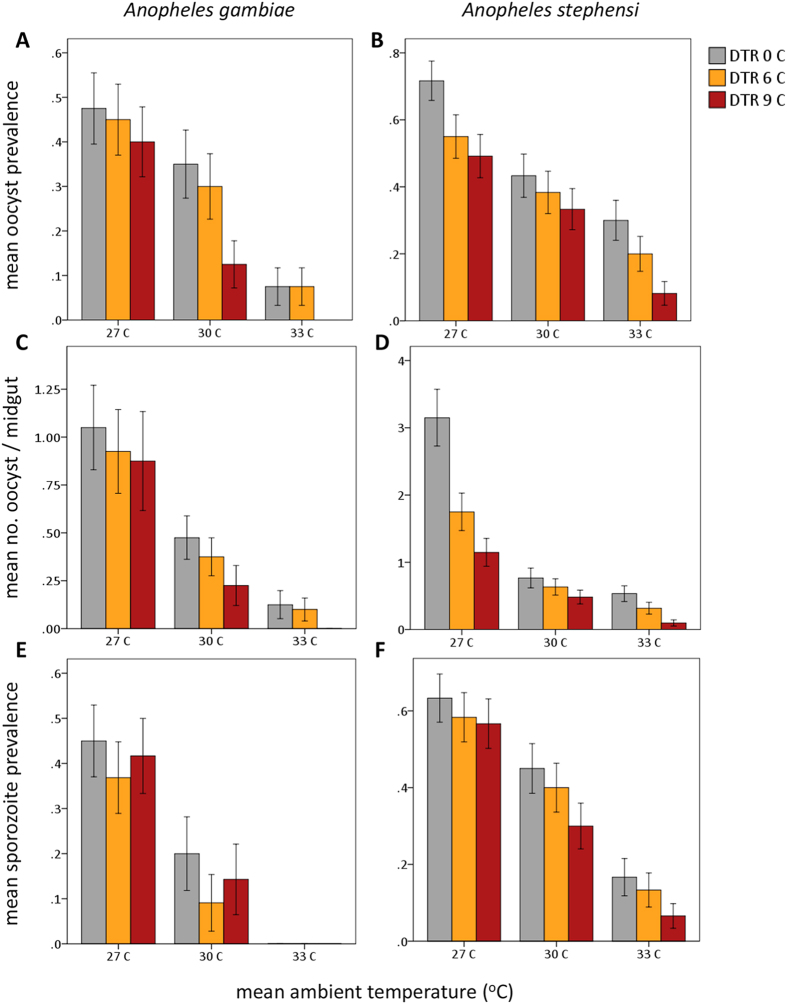
The effects of increasing mean ambient temperature and diurnal temperature range (DTR 0 C, grey bars; DTR 6 C, orange bars; and DTR 9 C, red bars) on measures of vector competence for *Anopheles gambiae* (left panel) and *An. stephensi* (right panel): mean oocyst prevalence (**A**,**B**), oocyst burdens (**C**,**D**), and sporozoite prevalence (**E**,**F**). Bars represent standard errors around the mean.

**Figure 2:**
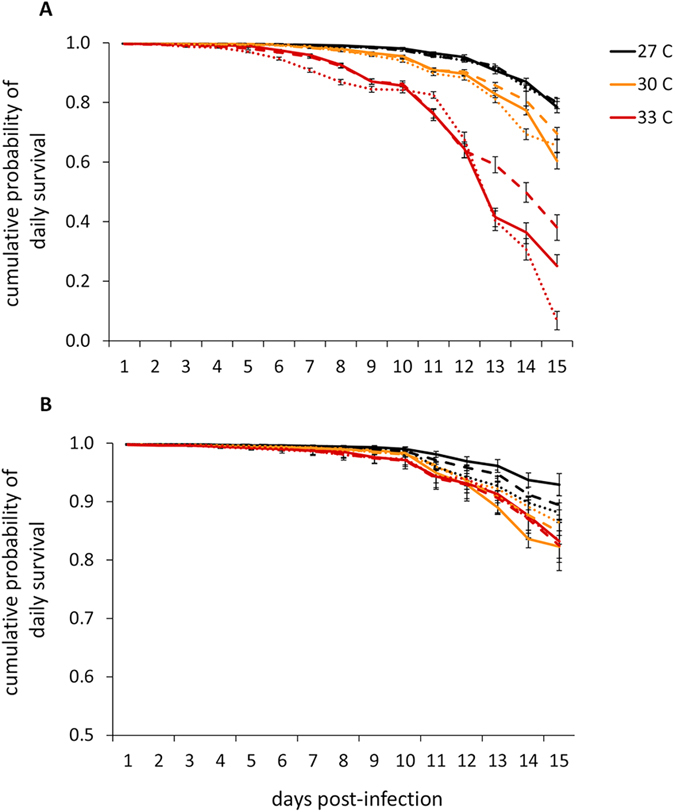
Increasing mean ambient temperature (27 °C, black lines; 30 °C, orange lines; 33 °C, red lines) decreases the cumulative probability of daily survival for both *Anopheles gambaie* (**A**) and *An. stephensi* (**B**). Variation in diurnal temperature range (DTR 0 C, solid lines; DTR 6 C, hashed lines; DTR 9 C, dotted lines) differentially affects the cumulative probability of daily mosquito survival for each vector species.

**Figure 3:**
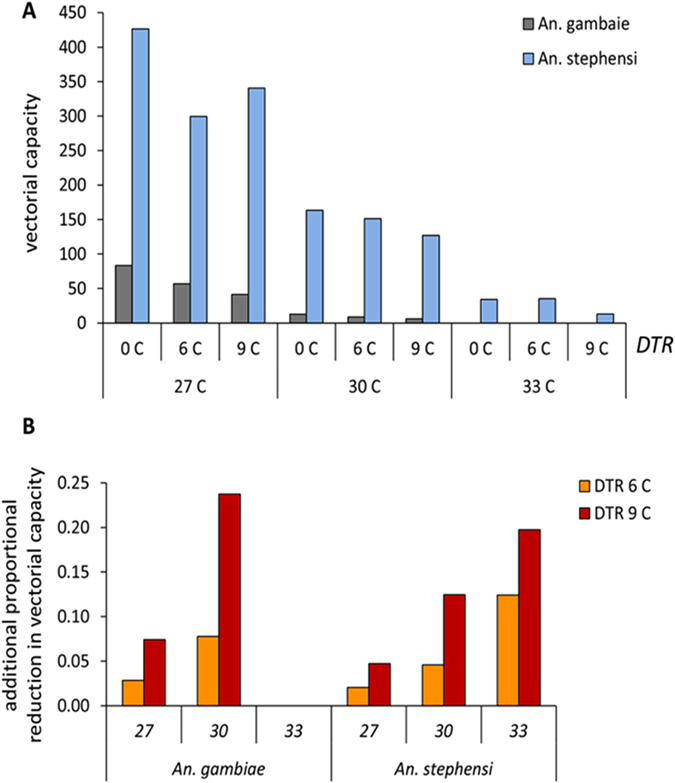
The effects of temperature and diurnal temperature range (DTR) on vectorial capacity. (**A**) Increasing mean ambient temperature (27, 30 and 33 °C) and DTR of (0, 6, 9 °C) decreases the vectorial capacity of both *Anopheles gambiae* (grey bars) and *An. stephensi* (blue bars). (**B**) When rate summation is used to estimate the predicted additional effects of DTR on biting rate and parasite development, the proportional reduction in vectorial capacity increases with mean ambient temperature and temperature variation. No predictions are available for *An. gambiae* at 33 °C as vectorial capacity is already zero without additional effects of DTR.

**Table 1:** The results of generalized mixed effects models for investigating the effects of variation in mean ambient temperature and diurnal temperature fluctuation on measures of vector competence for both *Anopheles gambiae* and *An. stephensi*.

	*Anopheles gambiae*									*Anopheles stephensi*								
	oocyst prevalence			oocyst intensity			sporozoite prevalence			oocyst prevalence			oocyst intensity			sporozoite prevalence		
**Factors**	F	d.f.	p	F	d.f.	p	F	d.f.	p	F	d.f.	p	F	d.f.	p	F	d.f.	p
intercept	72.07	8	<0.0001	63.93	8	<0.0001	28.90	8	<0.0001	31.23	8	<0.0001	23.39	8	<0.0001	9.64	8	<0.0001
temperature	37.96	2	<0.0001	11.14	2	0.004	14.38	1	<0.0001	57.04	2	<0.0001	64.20	2	<0.0001	36.73	2	<0.0001
DTR	6.41	2	0.041	0.33	2	0.847	1.75	2	0.417	14.56	2	<0.0001	16.77	2	<0.0001	2.94	2	0.052
temperature x DTR	–	–	–	–	–	–	–	–	–	–	–	–	6.43	4	0.001	–	–	–

Dashed lines indicate non-significant effects that were not included in the final model.

## References

[b1] Parham P. E. . Climate, environmental and socio-economic change: weighing up the balance in vector-borne disease transmission. Philos. T. Roy. Soc. B. 370, 1–17, 10.1098/rstb.2013.0551 (2015). PMC4342957 25688012

[b2] Mordecai E. A. . Optimal temperature for malaria transmission is dramatically lower than previously predicted. Ecol. Lett. 16, 22–30, 10.1111/ele.12015 (2013). 23050931

[b3] Siraj A. S. . Altitudinal changes in malaria incidence in highlands of Ethiopia and Colombia. Science 343, 1154–1158, 10.1126/science.1244325 (2014). 24604201

[b4] Pascual M., Ahumada J. A., Chaves L. F., Rodó X. & Bouma M. Malaria resurgence in the East African highlands: temperature trends revisited. Proc. Natl. Acad. Sci. USA 103, 5829–5834, 10.1073/pnas.0508929103 (2006). 16571662 PMC1416896

[b5] Paaijmans K. P., Read A. F. & Thomas M. B. Understanding the link between malaria risk and climate. Proc. Natl. Acad. Sci. USA 106, 13844–13849, 10.1073/pnas.0903423106 (2009). 19666598 PMC2720408

[b6] Paaijmans K. P. . Influence of climate on malaria transmission depends on daily temperature variation. Proc. Natl. Acad. Sci. USA 107, 15135–15139, 10.1073/pnas.1006422107 (2010). 20696913 PMC2930540

[b7] Paaijmans K. P. . Downscaling reveals diverse effects of anthropogenic climate warming on the potential for local environments to support malaria transmission. Clim. Change 125, 479–488 (2014).

[b8] Pachauri R. K. . Climate change 2014: synthesis report. Contribution of working groups I, II and III to the fifth assessment report of the Intergovernmental Panel on Climate Change. 151 (2014).

[b9] Caminade C. . Impact of climate change on global malaria distribution. Proc. Natl. Acad. Sci. USA 111, 3286–3291 (2014). 24596427 10.1073/pnas.1302089111 PMC3948226

[b10] Paaijmans K. P. & Thomas M. B. The influence of mosquito resting behaviour and associated microclimate for malaria risk. Malar. J. 10, 183, 183 10.1186/1475-2875-10-183 (2011). 21736735 PMC3146900

[b11] Cator L. J. . Characterizing microclimate in urban malaria transmission settings: a case study from Chennai, India. Malar. J. 12, 1–10, 84 10.1186/1475-2875-12-84 (2013). 23452620 PMC3599321

[b12] Murdock C. C., Moller-Jacobs L. L. & Thomas M. B. Complex environmental drivers of immunity and resistance in malaria mosquitoes. P. Roy. Soc. B-Biol Sci. 280, 10.1098/rspb.2013.2030 (2013). PMC3779341 24048159

[b13] Ferguson H. M. & Read A. F. Why is the effect of malaria parasites on mosquito survival still unresolved? Trends Parasitol. 18, 256–261, 10.1016/s1471-4922(02)02281-x (2002). 12036738

[b14] Vezilier J., Nicot A., Gandon S. & Rivero A. *Plasmodium* infection decreases fecundity and increases survival of mosquitoes. P. Roy. Soc. B-Biol Sci. 279, 4033–4041 (2012). 10.1098/rspb.2012.1394 PMC3427586 22859589

[b15] von Seidlein L. . Airflow attenuation and bed net utilization: observations from Africa and Asia. Malar. J. 11, 200 10.1186/1475-2875-11-200 (2012). PMC3441282 22704585

[b16] Dev V. & Sharma V. P. Anopheles mosquitoes - New insights into new malaria vectors (ed. Manguin S. ) Ch. 9, 239–271 (2013).

[b17] Paaijmans K. P., Imbahale S. S., Thomas M. B. & Takken W. Relevant microclimate for determining the development rate of malaria mosquitoes and possible impliations of climate change. Malar. J. 9, 196 (2010). 20618930 10.1186/1475-2875-9-196 PMC2912924

[b18] Paaijmans K. P. . Temperature variation makes ectotherms more sensitive to climate change. Glob. Change Biol. 19, 2373–2380 (2013). 10.1111/gcb.12240 PMC3908367 23630036

[b19] Christiansen-Jucht C., Parham P., Saddler A., Koella J. & Basanez M.-G. Temperature during larval development and adult maintenance influences the survival of *Anopheles gambiae* s.s. Parasit. Vectors 7, 489 (2014). 25367091 10.1186/s13071-014-0489-3 PMC4236470

[b20] Moller-Jacobs L., Murdock C. & Thomas M. Capacity of mosquitoes to transmit malaria depends on larval environment. Parasit. Vectors 7, 593 (2014). 25496502 10.1186/s13071-014-0593-4 PMC4273441

[b21] Sternberg E. D. & Thomas M. B. Local adaptation to temperature and the implications for vector-borne diseases. Trends Parasitol. 30, 115–122, 10.1016/j.pt.2013.12.010 (2014). 24513566

[b22] Blanford S., Read A. F. & Thomas M. B. Thermal behaviour of Anopheles stephensi in response to infection with malaria and fungal entomopathogens. Malar. J. 8, 1–9 (2009). 19379519 10.1186/1475-2875-8-72 PMC2683858

[b23] Kirby M. J. & Lindsay S. W. Responses of adult mosquitoes of two sibling species, *Anopheles arabiensis* and *A. gambiae* s.s. (Diptera: Culicidae), to high temperatures. Bull. Entomol. Res. 94, 441–448 (2004). 15385063 10.1079/ber2004316

[b24] Upadhyayula S. M., Mutheneni S. R., Chenna S., Parasaram V. & Kadiri M. R. Climate drivers on malaria transmission in Arunachal Pradesh, India. PLoS ONE 10, e0119514 (2015). 25803481 10.1371/journal.pone.0119514 PMC4372434

[b25] MacLeod D. A., Jones A., Di Giuseppe F., Caminade C. & Morse A. P. Demonstration of successful malaria forecasts for Botswana using an operational seasonal climate model. Environ. Res. Lett. 10, 044005 (2015).

[b26] Couret J. & Benedict M. A meta-analysis of the factors influencing development rate variation in *Aedes aegypti* (Diptera: Culicidae). BMC Ecol. 14, 3 (2014). 24495345 10.1186/1472-6785-14-3 PMC3916798

[b27] Couret J. Meta-analysis of factors affecting ontogenetic development rate in the *Culex pipiens* (Diptera: Culicidae) complex. Environ. Entomol. 42, 614–626 (2013). 23905724 10.1603/EN12248

[b28] Gething P. . Modelling the global constraints of temperature on transmission of Plasmodium falciparum and P. vivax. Parasit. Vectors 4, 92 (2011). 21615906 10.1186/1756-3305-4-92 PMC3115897

[b29] Ruybal J. E., Kramer L. D. & Kilpatrick A. M. Geographic variation in the response of *Culex pipiens* life history traits to temperature. Parasit. Vectors 9, 116 (2016). 26928181 10.1186/s13071-016-1402-z PMC4772444

[b30] Dell A. I., Pawar S. & Savage V. M. The thermal dependence of biological traits. Ecology 94, 1205–1206 (2013).

[b31] Kellermann V. . Upper thermal limits of *Drosophila* are linked to species distributions and strongly constrained phylogenetically. Proc. Natl. Acad. Sci. USA 109, 16228–16233 (2012). 22988106 10.1073/pnas.1207553109 PMC3479592

[b32] Araujo M. B. . Heat freezes niche evolution. Ecol. Lett. 16, 1206–1219 (2013). 23869696 10.1111/ele.12155

[b33] Stratman-Thomas W. K. The Influence of Temperature on *Plasmodium vivax*. Am. J. Trop. Med. Hyg 20, 703–715 (1940).

[b34] Smith D. L. & McKenzie F. E. Statics and dynamics of malaria infection in *Anopheles* mosquitoes. Malar. J. 3, 13 (2004). 15180900 10.1186/1475-2875-3-13 PMC449722

[b35] Meyrowitsch D. W. . Is the current decline in malaria burden in sub-Saharan Africa due to a decrease in vector population? Malar. J. 10, 1475–2875 (2011). 10.1186/1475-2875-10-188 PMC3160426 21752273

