# Supplementary material for: Malaria transmission potential could be reduced with current and future climate change
Source: Sci Rep. 2016 Jun 21;6:27771. doi: 10.1038/srep27771 (PMC4914975; doi:10.1038/srep27771)
Supplement: Supplementary Information [file srep27771-s1.doc]

**Supplementary Information Tables**

**Title:** Malaria transmission potential could be reduced with current and future climate change

**Authors**: C.C. Murdock, E.D. Sternberg, M.B. Thomas

**Table S1.** Significant pair-wise comparisons for temperature and diurnal temperature range effects on measures of vector competence for *Anopheles gambiae*. Dashes represent non-significant pair-wise comparisons.

|  | **oocyst prevalence** | | **oocyst intensity** | | **sporozoite prevalence** | |
| --- | --- | --- | --- | --- | --- | --- |
|  | *pair-wise*  *comparison* | *p* | *pair-wise*  *comparison* | *p* | *pair-wise*  *comparison* | *p* |
| *temperature* | 27 C vs. 30 C | 0.0002 | 27 C vs. 30 C | 0.0002 | 27 C vs. 30 C | 0.0002 |
|  | 27 C vs. 33 C | <0.0001 | - | - | 27 C vs. 33 C | <0.0001 |
|  | 30 C vs. 33 C | <0.0001 | - | - | - | - |
| *DTR* | DTR: 0 C vs. 9 C | <0.0001 | - | - | - | - |
|  | DTR: 6 C vs. 9 C | <0.0001 | - | - | - | - |

**Table S2.** Significant pair-wise comparisons for temperature and diurnal temperature range effects on measures of vector competence for *Anopheles stephensi*. Dashes indicate non-significant pair-wise comparisons.

|  | **oocyst prevalence** | | **oocyst intensity** | | **sporozoite prevalence** | |
| --- | --- | --- | --- | --- | --- | --- |
| **main effects** | *pair-wise*  *comparison* | *p* | *pair-wise*  *comparison* | *p* | *pair-wise*  *comparison* | *p* |
| *temperature* | 27 C vs. 30 C | <0.0001 | 27 C vs. 30 C | <0.0001 | 27 C vs. 30 C | <0.0001 |
|  | 27 C vs. 33 C | <0.0001 | 27 C vs. 33 C | <0.0001 | 27 C vs. 33 C | <0.0001 |
|  | 30 C vs. 33 C | <0.0001 | - | - | 30 C vs. 33 C | <0.0001 |
| *DTR* | DTR: 0 C vs. 9 C | 0.027 | - | - | - | - |
|  | DTR: 0 C vs. 9 C | <0.0001 | DTR: 0 C vs. 9 C | 0.003 | DTR: 0 C vs. 9 C | 0.036 |
| **temperature x DTR** | | |  |  |  |  |
| *27 C* | - | - | DTR: 0 C vs. 6 C | <0.0001 | - | - |
|  | - | - | DTR: 0 C vs. 9 C | <0.0001 | - | - |
| *DTR 0 C* | - | - | 27 C vs. 30 C | <0.0001 | - | - |
|  | - | - | 27 C vs. 33 C | <0.0001 | - | - |
| *DTR 6 C* | - | - | 27 C vs. 30 C | 0.0002 | - | - |
|  | - | - | 27 C vs. 33 C | 0.007 | - | - |

**Table S3.** Results from generalized linear mixed effects interval censor survival analysis on the effects of variation in infection status, mean ambient temperature, diurnal temperature range on the daily probability of mosquito survival. Dashed lines indicate non-significant factors and interactions not included in the final model.

|  | ***Anopheles gambiae*** | | | ***Anopheles stephensi*** | | |
| --- | --- | --- | --- | --- | --- | --- |
| **Factors** | *F* | *d.f.* | *p* | *F* | *d.f.* | *p* |
| *intercept* | 94.96 | 25 | <0.0001 | 93.76 | 25 | <0.0001 |
| *infection status* | 6.17 | 1 | 0.013 | 4.86 | 1 | 0.028 |
| *temperature* | 473.95 | 2 | <0.0001 | 118.00 | 2 | <0.0001 |
| *DTR* | 14.28 | 2 | <0.0001 | 4.04 | 2 | 0.018 |
| *days post-infection* | 160.12 | 14 | <0.0001 | 156.43 | 14 | <0.0001 |
| *infection status x temperature* | 3.77 | 2 | 0.024 | - | - | - |
| *infection status x DTR* | - | - | - | 6.12 | 2 | 0.002 |
| *temperature x DTR* | 3.29 | 4 | 0.011 | 2.54 | 4 | 0.038 |

**Table S4.** Pair-wise comparisons of temperature and diurnal temperature range effects on the daily probability of survival for both *Anopheles gambiae* and *An. stephensi*. Dashed lines indicate non-significant pair-wise comparisons.

|  | ***Anopheles gambiae*** | | ***Anopheles stephensi*** | |
| --- | --- | --- | --- | --- |
|  | *pair-wise comparison* | *p* | *pair-wise comparison* | *p* |
| *DTR 0 C* | 27 C vs. 30 C | <0.0001 | 27 C vs. 30 C | <0.0001 |
|  | 27 C vs. 33 C | <0.0001 | 27 C vs. 33 C | <0.0001 |
|  | 30 C vs. 33 C | <0.0001 | - | - |
| *DTR 6 C* | 27 C vs. 30 C | <0.0001 | 27 C vs. 30 C | <0.0001 |
|  | 27 C vs. 33 C | <0.0001 | 27 C vs. 33 C | <0.0001 |
|  | 30 C vs. 33 C | <0.0001 | 30 C vs. 33 C | <0.0001 |
| *DTR 9 C* | 27 C vs. 30 C | <0.0001 | 27 C vs. 30 C | 0.033 |
|  | 27 C vs. 33 C | <0.0001 | 27 C vs. 33 C | <0.0001 |
|  | 30 C vs. 33 C | <0.0001 | 30 C vs. 33 C | 0.002 |
| *27 C* | DTR 0 C vs. DTR 6 C | - | DTR 0 C vs. DTR 6 C | <0.0001 |
|  | DTR 0 C vs. DTR 9 C | - | DTR 0 C vs. DTR 9 C | <0.0001 |
|  | DTR 6 C vs. DTR 9 C | - | DTR 6 C vs. DTR 9 C | - |
| *30 C* | DTR 0 C vs. DTR 6 C | - | DTR 0 C vs. DTR 6 C | 0.036 |
|  | DTR 0 C vs. DTR 9 C | - | DTR 0 C vs. DTR 9 C | 0.005 |
|  | DTR 6 C vs. DTR 9 C | 0.003 | DTR 6 C vs. DTR 9 C | - |
| *33 C* | DTR 0 C vs. DTR 6 C | - | DTR 0 C vs. DTR 6 C | - |
|  | DTR 0 C vs. DTR 9 C | 0.002 | DTR 0 C vs. DTR 9 C | - |
|  | DTR 6 C vs. DTR 9 C | <0.0001 | DTR 6 C vs. DTR 9 C | - |

**Table S5.** Pair-wise comparisons of infection effects mediated by temperature and diurnal temperature range on the daily probability of survival for both *Anopheles gambiae* and *An. stephensi*. Dashes indicate non-significant pair-wise comparisons.

| ***Infection Status x Temperature*** | |  | ***Infection Status x DTR*** | |  |
| --- | --- | --- | --- | --- | --- |
|  | ***Anopheles gambiae*** | |  | ***Anopheles stephensi*** | |
| *treatment* | *pair-wise comparison* | *p* | *treatment* | *pair-wise comparison* | *p* |
| *bloodfed controls* | 27 C vs. 30 C | <0.0001 | *bloodfed controls* | DTR 0 C vs. DTR 6 C | 0.001 |
|  | 27 C vs. 33 C | <0.0001 |  | DTR 0 C vs. DTR 9 C | 0.002 |
|  | 30 C vs. 33 C | <0.0001 |  | DTR 6 C vs. DTR 9 C | - |
| *P. falciparum* | 27 C vs. 30 C | <0.0001 | *P. falciparum* | DTR 0 C vs. DTR 6 C | - |
|  | 27 C vs. 33 C | <0.0001 |  | DTR 0 C vs. DTR 9 C | - |
|  | 30 C vs. 33 C | <0.0001 |  | DTR 6 C vs. DTR 9 C | - |
| *27 C* | *bloodfed vs. falciparum* | - | *DTR 0 C* | *bloodfed vs. falciparum* | 0.001 |
| *30 C* | *bloodfed vs. falciparum* | - | *DTR 6 C* | *bloodfed vs. falciparum* | - |
| *33 C* | *bloodfed vs. falciparum* | <0.0001 | *DTR 9 C* | *bloodfed vs. falciparum* | - |

**Table S6.** Parameters were set at the following values for each fluctuation around means 27^o^C, 30^o^C, & 33^o^C.

|  |  | **DTR 6 C** |  |  | **DTR 9 C** |  |
| --- | --- | --- | --- | --- | --- | --- |
| **Parameters** | **27 C** | **30 C** | **33 C** | **27 C** | **30 C** | **33 C** |
|  |  |  |  |  |  |  |
| *T_min_* | 24 C | 27 C | 30 C | 22.5 C | 25.5 C | 28.5 C |
| *T_max_* | 30 C | 33 C | 36 C | 31.5 C | 34.5 C | 37.5 C |
| *t_rise_* | 6:00 AM | 6:00 AM | 6:00 AM | 6:00 AM | 6:00 AM | 6:00 AM |
| *t_set_* | 6:00 PM | 6:00 PM | 6:00 PM | 6:00 PM | 6:00 PM | 6:00 PM |
| *D* | 12 hrs | 12 hrs | 12 hrs | 12 hrs | 12 hrs | 12 hrs |
| *N* | 12 hrs | 12 hrs | 12 hrs | 12 hrs | 12 hrs | 12 hrs |
| *P* | 1.5 hrs | 1.5 hrs | 1.5 hrs | 1.5 hrs | 1.5 hrs | 1.5 hrs |
| *τ* | 4 | 4 | 4 | 4 | 4 | 4 |

**Supplementary Information Figures**

**Figure S1.**

**A**


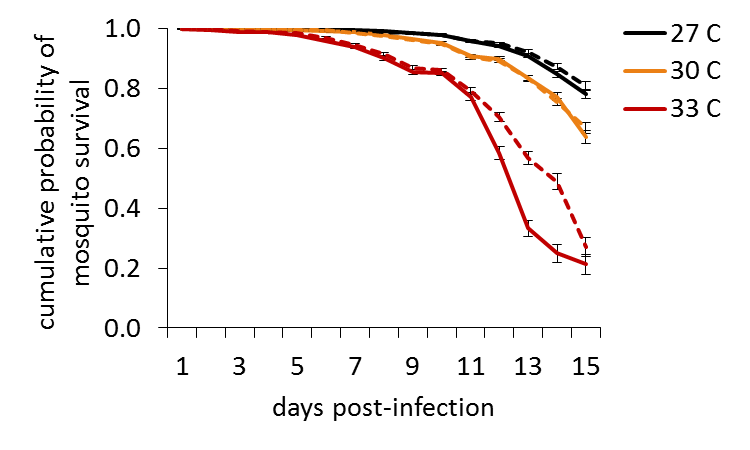


**B**


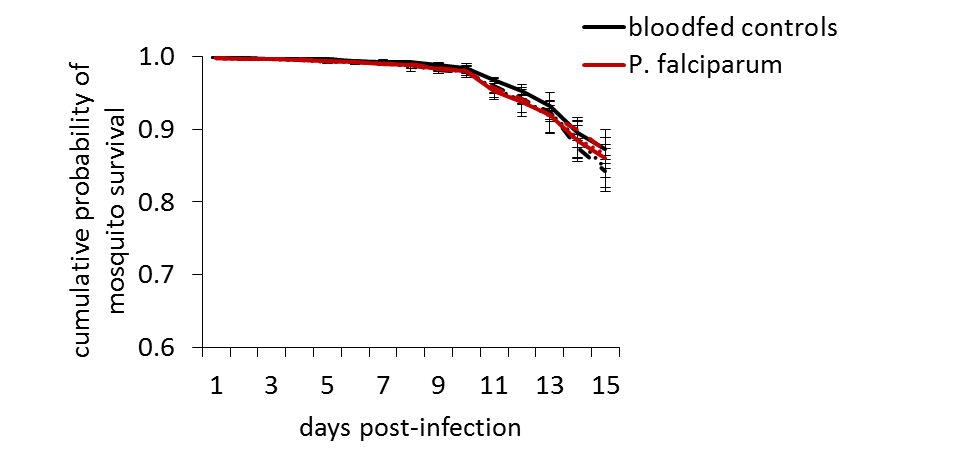


**A** The effects of infection status on *An. gambiae* daily survival are mediated by mean ambient temperature. Solid lines and dotted lines represent bloodfed control and *P. falciparum* infectious mosquitoes, respectively. **B** The effects of infection status on *An. stephensi* daily survival are mediated by DTR. Solid lines, hashed lines, and dotted lines represent DTR 0 C, DTR 6 C, and DTR 9 C, respectively. For both A and B bars around each mean represent standard errors.

**Figure S2.**

**A**

**B**

The effect of house type on indoor temperature in **A** Tanzania (year-long sampling period) and **B** Gambia (four month sampling period). Methods for collecting these data are published in [39]. The solid line is the daily mean temperature for each house, averaged by house type. The dashed and dotted lines represent daily maximums and minimums, respectively, again averaged by house type. Daily mean temperature and daily minimum temperature does not change substantially with housing structure type, however brick houses with metal roofs (red lines) experienced higher daily maximum temperatures than mud homes with thatch roofs (black lines).

**Figure S3. Experimental Design**

After being fed an infectious *Plasmodium falciparum*) or uninfected bloodmeal on day three post-emergence, we randomly distributed approximately 150 *Anopheles gambiae* (*AG*) and *An. stephensi* (*AS*) from each infection regime across three mean ambient temperatures (27^o^C, 30^o^C, and 33^o^C) and three diurnal temperature ranges (DTR 0^o^C, DTR 6^o^C, and DTR 9^o^C). We replicated this experiment fully two and three times for *An. gambiae* and *An. stephensi*, respectively.

**Figure S4.**

The following parameters were set at these values for each fluctuation around means 27^o^C, 30^o^C, and 33^o^C. Each temperature treatment is represented above, with 33^o^C DTR 0^o^C, 33^o^C DTR 6^o^C, and 33^o^C DTR 9^o^C represented by grey, orange, and red lines, respectively.

**Supplementary Information Methods**

***Parton-Logan Model***

The Parton-Logan model, characterized by a sinusoidal progression during the daytime and a decreasing exponential curve during the night, is a good representation of both the phase and form of natural diurnal temperature rhythms.


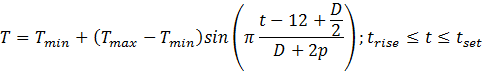


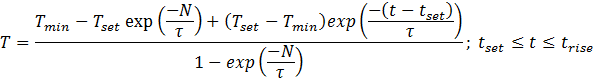


where *T_min_* and *T_max_* (^o^C) are the minimum and maximum daily air temperatures, *t* (hrs) the time, *D* (hrs) the day length, *p* (1.5 hr) the time duration between solar noon and *T_max_*, *t_rise_* (hrs) the time of sunrise, *t_set_* (hrs) the time of sunset, *T_set_* (^o^C) the temperature at sunset, *N* (hrs) the duration of the night, and *τ* the nocturnal time constant (Table S5). We then programmed the diurnally fluctuating incubators with temperatures generated from the above model across various time points in a 24 hr period of time (Figure S4).

***Quantifying Vector Competence***

To quantify oocyst prevalence and intensity, or sporozoite prevalence, we aspirated 20 mosquitoes into 95% ethanol from each *P. falciparum* infected treatment group and dissected their midguts (day 7 post-infection) or salivary glands (day 15 post-infection), respectively, in 1X phosphate-buffered saline solution under a standard dissecting scope. Using a compound microscope, we noted whether midguts or salivary glands were infected or uninfected, and counted the number of *Plasmodium* oocysts that had established in each infected midgut.

***Statistical Analyses***

We fit binomial distributions (logit function), a linear distribution, and a binomial distribution (complementary log-log function) for the models with oocyst / sporozoite prevalence, oocyst intensity, and daily mosquito survival as response variables, respectively. Variance structures assigned to our random factor, replicate, for each model were chosen based on those structures that yielded the model with the lowest AIC score. We assessed goodness of fit of all statistical models through log likelihood values and model residuals. Covariates included in mixed effects GZLMs were centred on their grand mean, and adjusted Bonferroni post-hoc tests were used to identify significant pair-wise comparisons.
